# The Characteristics of Chancres of the Cervix Uteri

**Published:** 1882-04

**Authors:** 


### ORIGINAL TRANSLATIONS AND ABSTRACTS.

### THE CHARACTERISTICS OF CHANCRES OF THE CERVIX UTERI.

Dr. Mracek (*Arch. f. Derm. und Syph.*, 1, 1881) reports twenty-four cases of initial lesion of syphilis occurring on the vaginal portion of the cervix. In most cases the lesion was upon the anterior lip and on the os. There seems to be a greater liability in women who have borne children. It is not probable that syphilis can occur without an initial lesion.

The earliest stage of the lesion does not present the characteristic appearances—induration, sharp, livid border, diphtheritic deposit—which the ulcer assumes at a later period. Several tubercles are often found in the vicinity of the lesion. Inguinal glandular swellings and papulæ about the labia and vestibule are not infrequent. If the consecutive symptoms of syphilis are already present the vaginal portion is firm, enlarged, the tubercles have ulcerated bases covered with bleeding granulations. If the initial lesion is situated in the cervical canal, it can of course only be discovered after ulceration or sloughing of the os.

The progress of chancre of the cervix may be almost entirely free from symptoms. It is generally so slow that the primary lesion may still be present when the secondary symptoms have already disappeared. Cicatrization of the sore proceeds slowly and may be frequently interrupted or retrograde. Stenosis and atresia of the cervix have been observed in cases. The hypertrophy and induration of the cervical tissue may cause dystocia.

The treatment is principally local-vaginal irrigation, three or four times a day, with a one per cent solution of potassium chlorate or carbolic acid ; tampons saturated with solutions of iodine, chloride of iron, and iodoform ; if much infiltration of the cervix exists, painting with tincture of iodine or bichloride of mercury solution (1–10). Exuberant granulations are touched with solid silver nitrate. Stenosis of the cervix is remedied by the insertion of tents. The usual constitutional treatment is pursued.

